# Residual Effects of Sleep Medications Are Commonly Reported and Associated with Impaired Patient-Reported Outcomes among Insomnia Patients in the United States

**DOI:** 10.1155/2015/607148

**Published:** 2015-12-09

**Authors:** Timothy Fitzgerald, Jeffrey Vietri

**Affiliations:** ^1^Merck & Co., Whitehouse Station, NJ 08889, USA; ^2^Kantar Health, 20121 Milan, Italy

## Abstract

*Study Objective*. To measure the association of symptoms attributed to residual effects of sleep medication (e.g., drowsiness, difficulty concentrating, and impaired memory) on self-reported functioning and satisfaction with these medications.* Methods*. Individuals using prescription medications for insomnia were invited to complete an Internet-based survey. Respondents were compared according to the presence of self-reported residual effects; relationships between severity of these effects and outcomes were modeled using regression. Measures included the Brief Insomnia Questionnaire, Work Productivity and Activity Impairment Questionnaire, and SATMED-Q. Subgroup analyses were conducted with patients aged ≥65 years. Approximately 80% reported experiencing ≥1 residual effect. The severity of residual effects was associated with increased residual effect-related work impairment, including absenteeism (RR = 1.46, *p* < 0.001), presenteeism (RR = 1.12, *p* < 0.001), overall work impairment (RR = 1.13, *p* < 0.001), and nonwork activity impairment (RR = 1.11, *p* < 0.001). More severe residual symptoms were also associated with increased difficulty in home management (Beta = .31, *p* < 0.001), ability to work (Beta = .31, *p* < 0.001), social relationships, (Beta = .32, *p* < 0.001), close personal relationships (Beta = .30, *p* < 0.001), and lower medication satisfaction (Beta = −.37, *p* < 0.001).* Conclusions*. Individuals using medications for insomnia commonly experience symptoms considered as residual effects, and these symptoms are associated with greater interference of sleep-related problems at work, at home, and with social relationships.

## 1. Introduction

Insomnia is a debilitating condition that accompanies several sleep, medical, and psychiatric disorders. It is diagnosed via subjective reports of persistent difficulty falling asleep, staying asleep, and/or experiencing sleep of poor quality. Insomnia confers significant daytime symptoms such as fatigue, low energy, impaired cognitive functioning, mood disturbance, and perceptions of decreased global functioning. In fact, daytime symptoms are those which most frequently lead patients to seek treatment [1]. Acute insomnia regularly occurs with life events or sleep schedule changes. For some, however, insomnia becomes unrelenting and chronic. Overall, insomnia has become a prevalent and costly public health concern, associated with long-term effects on functioning and quality of life.

Approximately 25% of U.S. adults report dissatisfaction with their sleep, 10–15% report insomnia symptoms, and 6–10% have an insomnia disorder [2]. Population-based longitudinal data show nearly 70% of patients with baseline insomnia also report insomnia one year later, and 50% of those with baseline insomnia continue to report insomnia up to three years later [3, 4]. In fact, insomnia has become one of the most prevalent complaints in the primary care setting [5]. Moreover, high rates of comorbidity between insomnia and medical/psychiatric disorders have been described. Odds ratios reported by the 2002 U.S. National Health Interview Survey and the U.S. National Comorbidity Survey showed those with insomnia to be twice as likely to present with congestive heart failure and up to five times more likely to present with a psychiatric disorder [6, 7]. Further, insomnia is strongly associated with hypertension and pain conditions, as well as greater risk of mortality, specifically in older adults [8–10].

Treatment can consist of behavioral/psychological interventions and/or pharmacotherapy. In general, it is considered that primary insomnia and secondary insomnia respond to both pharmacotherapy and behavioral/psychological intervention [11, 12]. Common agents to treat insomnia include over-the-counter agents (OTCs, antihistamines, melatonin, and herbal preparations), prescription hypnotic drugs (benzodiazepines, BzRAs, chronobiotic agents, and low-dose doxepin hydrochloride), and other prescription agents not specifically indicated for insomnia (antidepressants, antipsychotics, and anticonvulsants) [13]. However, sleep-promoting agents can produce adverse effects, particularly in the elderly [13]. Residual sleep medication effects have the potential to interfere with quality of life and include drowsiness, difficulty concentrating, headaches, nausea, dry mouth, oversleeping, and nightmares. Memory can also be affected, including impaired short-term memory and periods of amnesia reported in the literature [14–16].

Of the hypnotics, benzodiazepines and nonbenzodiazepine hypnotics with longer half-lives tend to produce residual impairment or “hang over,” particularly with middle-of-the-night dosing and regular use [17, 18]. Residual effects of hypnotics include sedation, cognitive impairment, motor incoordination, ataxia, dizziness, and gastrointestinal upset. In the elderly, the use of sedating drugs is dangerously associated with increased fall risk [19]. Meanwhile, the use of some antidepressants to treat insomnia has been associated with increased suicidal ideation, increased mania/hypomania in patients with bipolar disorder, and exacerbation of restless legs syndrome [20]. Further, the use of anticonvulsants (e.g., pregabalin) can produce daytime sedation, dizziness, and cognitive impairment [13]. Finally, the use of antipsychotics has been correlated with exacerbation of restless legs syndrome and increased mortality, particularly in elderly individuals [11]. From the standpoint of concern for public safety, insomnia treatments have been shown to impair next-day driving and increase the risk of motor vehicle accidents, particularly in women [21].

As above, older patients are particularly vulnerable to residual sleep medication effects. Meta-analysis of patients aged 60 years and older, who were free of other psychiatric disorders (*N* = 2,417), showed significantly higher odds of adverse cognitive events, adverse psychomotor effects, and daytime fatigue when patients used any hypnotic sedative, compared with placebo [22]. A large (*N* = 15,528) study of nursing home patients (mean age = 81), with hip fracture documented in Medicare Part A and Part D fee-for-service claims, showed elevated risk of hip fracture among users of a nonbenzodiazepine hypnotic sleep drug. Effects were particularly strong in new users [23].

Ultimately, residual sleep medication effects are associated with impaired functioning and lowered quality of life in insomnia patients and confer safety risks for both the patients and the public. Moreover, the literature reports older patients to be at heightened risk for adverse side effects [13]. Little research, however, has characterized the impact of adverse side effects across multiple domains of functioning in the same group of patients—particularly in the elderly. The current study was conducted to collect information on the relationship between self-reported residual effects of prescription sleep medications and patient-reported outcomes. A secondary objective of the study was to describe the relationship between these symptoms and outcomes in users of these medications aged 65 years and older, since, as described above, these patients may be particularly vulnerable to residual effects [13].

## 2. Materials and Methods

The present study was a cross-sectional survey of current and former users of prescription medications for sleep reporting a diagnosis of insomnia (*N* = 2,250). For the purpose of estimating the burden of residual effects, only those currently using a sleep medication (*n* = 1,605) were included. Those reporting residual sleep medication effects were compared to those reporting no residual effects, and the relationship between severity of residual effects and outcomes was modeled using regression. Subgroup analyses were conducted with patients aged 65 years or older due to reported vulnerability to adverse sleep medication effects [13].

Respondents were recruited primarily from previous respondents to the U.S. National Health and Wellness Survey (NHWS). The NHWS is a cross-sectional survey administered via the Internet to a sample of adults (18 years and older) who were identified through a web-based consumer survey panel. Members of the panel are recruited through opt-in emails, coregistration with other panels, e-newsletter campaigns, and online banner placements. All panelists explicitly agreed to become panel members, registered through unique email addresses, and completed in-depth demographic registration profiles. Invitations to participate in the NHWS were sent using a random stratified sampling framework to ensure the final sample of NHWS participants is representative of the adult population in the U.S. according to the Current Population Survey (CPS) of the U.S. Census (United States Bureau of the Census, 2012) in terms of age, gender, and white/non-white racial identification. Because of the size of the target sample and inclusion criteria of this study, additional respondents were also identified through the Light Speed Research Ailment Panel, which is composed of general panel members who have self-identified as having certain medical conditions.

Only those who were aged 18 years or older, self-reported a diagnosis of insomnia, and did not self-report diagnosis for sleep-disordered breathing, narcolepsy, shift work disorder, parasomnia, or other sleep condition were included in the survey. For inclusion in the current analysis, respondents also had to report current use of a prescription medication for sleep.

### 2.1. Procedure

Respondents were interviewed through a self-administered, Internet-based questionnaire between December 5th and 18th, 2012. The protocol and questionnaire were reviewed and approved by Essex Institutional Review Board (Lebanon, New Jersey, USA) prior to recruitment of participants.

The presence and severity of residual effects were assessed through a series of items assessing to what extent the respondent experienced each of the nine side effects when they take their insomnia medication. These were feelings of drowsiness, difficulty concentrating, difficulty remembering, headaches, nausea, dry mouth, oversleeping, amnesia, and nightmares, using a Likert scale from 1 (None) to 5 (“Very Severe”). Drowsiness, difficulty concentrating, difficulty remembering, headaches, nausea, dry mouth, and oversleeping were also combined by summing the ratings of severity to provide an overall index of residual symptoms.

Respondents who indicated at least one residual symptom completed the Work Productivity and Activity Impairment, Specific Health Problem (WPAI-SHP) questionnaire [24]. The specific health problem was residual symptoms, and the term used in the instrument for residual symptoms was “next-day effects.” Respondents were informed that this term was meant to indicate any side effects they feel the day after taking the medication. Four subscales (absenteeism, presenteeism, overall work impairment, and activity impairment) were generated in the form of percentages, with higher values indicating greater impairment. Absenteeism represents the percentage of work time missed due to next-day effects of sleep medication in the past seven days, and presenteeism represents the percentage of impairment in the past seven days while at work. Overall work impairment represents the overall amount of impact to work productivity due to either absenteeism or presenteeism (since they are mutually exclusive) in the past seven days. Activity impairment represents the percentage of impairment experienced during daily activities in the past seven days. Only employed respondents provided data on absenteeism, presenteeism, and overall work impairment but all respondents who reported at least one residual symptom rated their activity impairment.

Information on sleep difficulties was assessed using the Brief Insomnia Questionnaire (BIQ [25]). Information collected in the BIQ included in this analysis was the number of nights out of the past 7 with sleep problems due to trouble falling asleep, trouble staying asleep, trouble waking too early, waking feeling tired or unrested, and nights with at least one of these problems. The degree to which the individual's sleep problems interfered with home management, ability to work, social relationships, and close personal relationships was also assessed in the BIQ using a modified version of the Sheehan Disability Scales. These are scored from 0 to 10, with higher numbers indicating greater interference. A “don't know” response is also provided, and those who selected this option were excluded from analysis of the relevant item.

*Insomnia Treatment History*. A variety of items were used to characterize the respondents' treatment history for insomnia. These include the year diagnosed with insomnia, type of diagnosing doctor, type of prescribing doctor, type of doctor currently managing insomnia, whether the respondent is currently seeing a healthcare provider for insomnia, and previous prescription medications taken for insomnia.

Satisfaction with current medication was measured by the SATMED-Q [26]. This scale includes a total of 17 items that measure treatment satisfaction across multiple domains, including the presence and interference caused by side effects, the efficacy of the medication, convenience and ease of use, impact of medicine on everyday life, the follow-up from the doctor, and the patient's overall opinion of the medicine.

Health characteristics and risk behaviors incorporated in the analysis included body mass index (BMI; underweight, normal weight, overweight, and obese), alcohol consumption (consume alcohol versus abstain from alcohol), cigarette smoking (current smoker versus nonsmoker), and whether the person reports exercising vigorously in the past 30 days. The severity of respondents' comorbid medication conditions was assessed using the Charlson comorbidity index (CCI [27]). The CCI is calculated by weighting the presence of the following conditions and summing the result: HIV/AIDS, metastatic tumor, lymphoma, leukemia, any tumor, moderate/severe renal disease, hemiplegia, diabetes, mild liver disease, ulcer disease, connective tissue disease, chronic pulmonary disease, dementia, cerebrovascular disease, peripheral vascular disease, myocardial infarction, and congestive heart failure. The greater the total index score, the greater the comorbidity burden on the patient. Because insomnia commonly presents with psychiatric disorders, self-reported psychiatric diagnoses were also included in the questionnaire, including alcoholism, anxiety, bipolar disorder, depression, fibromyalgia, and schizophrenia.

### 2.2. Analysis

Analyses were conducted both on the full sample (aged 18 and older) and among the portion of the sample aged 65 years and older. Initial analyses compared those experiencing no residual symptoms to those who experienced at least one residual symptom using chi-square test for categorical variables and independent-samples *t*-tests for continuous variables. The relationship between the severity of residual effects and outcomes was also analyzed using multivariable regression. The multivariable models adjusted for covariates to reduce the likelihood that observed effects of residual symptoms were due to confounding factors. Covariates included gender (male versus female), race/ethnicity, age (continuous), BMI (overweight, obese, and missing versus normal/underweight), household income, comorbidity burden according to CCI, and a variety of psychiatric illnesses, which were found to be associated with residual symptoms during review of bivariate analyses. Models of treatment satisfaction were conducted using the total score from the SATMED-Q and were conducted using maximum likelihood linear regression. Likewise, ratings of disability were approximately normally distributed and also analyzed using linear models. Models of impairments measured by the WPAI were conducted using generalized linear models (GLMs) specifying a negative binomial distribution and a log-link function. All analyses were conducted first in the full sample and repeated in the subsample aged 65 and older.

## 3. Results

Sample characteristics are presented in Table 1. Respondents were 52 years old on average, 78% were female and 87% were white. Most had their insomnia diagnosed and managed by a general practitioner. Psychiatric comorbidities were common among the sample, with approximately 50% reporting depression and approximately one-third of the sample reporting an anxiety disorder.

Approximately 80% of current users (1,274/1,605) indicated some level of residual symptoms. Those reporting residual symptoms were slightly younger on average, but otherwise there were few demographic characteristics that differed across the presence of residual symptoms (Table 2). However, health characteristics differed according to presence of residual symptoms, with anxiety, depression, schizophrenia, and fibromyalgia all more likely among those with residual symptoms relative to those without residual symptoms, while alcoholism and bipolar disorder were marginally more likely. Psychiatrists were more often the diagnosing and prescribing doctor for those with residual symptoms than those without. The burden of comorbid conditions as represented by the CCI did not differ according to residual symptoms.

Problems with sleep in the prior 7 nights were common in current users of sleep medications. The presence of residual symptoms was associated with one additional day waking up tired/unrested, but not with the number of nights out of the past 7 with trouble falling asleep, staying asleep, or waking before the alarm (Table 3). In contrast, the impact of poor sleep on functioning was greater among those with residual effects, however, as ratings of interference in home management, ability to work, social relationships, and close relationships were all significantly higher among those reporting residual effects. Likewise, those who experienced residual symptoms were less satisfied with their current sleep medication than those who did not experience any residual symptoms (69.2 versus 76.0, *p* < 0.001).

Correlational analyses confirmed that when present, severity of residual symptoms was associated with worse outcomes and lower satisfaction. All residual symptoms were significantly associated with greater ratings of work and activity impairment in bivariate correlations. Difficulty concentrating and drowsiness were particularly burdensome, which were correlated with work and activity impairment *r*_*s*_ = .46–.49. Likewise, the severity of difficulty concentrating (*r*_*s*_ = −.347, *p* < 0.001) and grogginess (*r*_*s*_ = −.366, *p* < 0.001) was most associated with (reduced) satisfaction with sleep medication (data not presented).

Regression analyses confirmed the association between residual symptoms and outcomes. The severity of residual symptoms was associated with lower satisfaction as measured by the SATMED-Q, (Beta = −.37, *p* < 0.001). The severity of residual symptoms was also associated with increased residual symptom-related work impairment, including absenteeism (RR = 1.46, 95% CI: 1.34–1.60, *p* < 0.001), presenteeism (RR = 1.12, 95% CI: 1.09–1.14, *p* < 0.001), overall work impairment (RR = 1.13, 95% CI: 1.10–1.15, *p* < 0.001), and impairment in nonwork activities (RR = 1.11, 95% CI: 1.10–1.13, *p* < 0.001). The severity of residual symptoms was also associated with increases in sleep-related interference on the four domains measured in the BIQ in the regression analyses; home management (Beta = .31, *p* < 0.001), ability to work (Beta = .31, *p* < 0.001), social relationships, (Beta = .32, *p* < 0.001), and close personal relationships (Beta = .30, *p* < 0.001) were all similarly affected.

Analysis of those aged 65 and older also revealed a high proportion (71%; 195 of 273) of current users reporting at least one residual symptom. As in the full sample, the rates of anxiety, depression, schizophrenia, and fibromyalgia were higher among those with residual symptoms (Table 4). Unlike the full sample, patients aged 65 or older with residual symptoms had higher CCI scores relative to those without residual symptoms.

Results of comparisons of sleep outcomes also mirrored those of the full sample (Table 5). The number of nights with different types of sleep problems were comparable across those with and without residual symptoms except for days waking up tired or unrested. Also consistent with the full sample, levels of disability due to sleep problems were elevated in those with residual symptoms relative to those without for all four domains measured. Those who experienced at least one residual symptom also had marginally lower satisfaction than those without any residual symptoms (74.7 versus 78.5, *p* = 0.057).

As in the full sample, the expected relationship between residual symptoms and satisfaction with sleep medication was seen in the correlations between satisfaction and ratings of individual residual symptoms (data not presented). Difficulty concentrating was most closely related to satisfaction (*r*_*s*_ = −.34, *p* < 0.001). Total residual symptoms and difficulty concentrating were most closely related to sleep medication-related impairment to nonwork activities (both *r*_*s*_ = .46, *p* < 0.001).

Regression results demonstrated that total residual symptoms were associated with lower satisfaction with current medication among those aged 65 years and older (Beta = −.37, *p* < 0.001). The severity of residual symptoms was also associated with increases in sleep-related interference on home management (Beta = .30, *p* < 0.001), social relationships (Beta = .26, *p* < 0.001), and close personal relationships (Beta = .27, *p* < 0.001). Total residual symptoms were also associated with impairment to nonwork activities on the WPAI (RR = 1.18, 95% CI: 1.11–1.25, *p* < 0.001).

## 4. Discussion

This study described the relationship between perceived residual sleep medication effects and a wide range of important outcomes for insomnia patients. This was the first study, to our knowledge, to describe the magnitude of the relationship between residual sleep medication effects and this large array of patient-reported outcomes, particularly in a single, large sample. Findings are particularly novel for the older patients, as the literature focuses primarily on what the residual effects are, rather than their correlates, for this demographic group.

Residual medication effects—such as feelings of being drowsy, groggy, or sluggish the next day, difficulty concentrating/remembering, or sleeping too much—were reported by approximately four out of every five individuals currently using prescription sleep medication. Overall, findings showed significant burden experienced by patients reporting residual sleep medication effects relative to those not reporting such effects.

Though patients with and without perceived residual effects suffered a similar number of nights with sleep problems (falling asleep, staying asleep, waking before the alarm, or any problem), the experience of residual effects was associated with an average of one more day per week of “unrestful sleep.” One potential explanation is that the residual effects of the sleep medication itself are responsible for the difference, though this is only speculation; the present analysis was not designed to identify the cause. Patients reporting residual effects were also less satisfied with their medications. Moreover, there were clear relationships between increasingly severe residual symptoms and decreased satisfaction, as well as increasingly severe residual symptoms and greater work and activity impairment, and greater sleep-related interference in home management, ability to work, and social relationships. Though respondents reporting residual effects indicated they experienced more psychiatric symptomatology and other comorbidities than those not experiencing such effects, the relationships between functioning and residual symptom severity remained significant after these and other relevant covariates were accounted for.

Analysis of older patients showed a similar pattern of relationships. Differences between those with and without residual symptoms were only marginal, but the correlation between increasingly severe residual symptoms and decreased satisfaction was of considerable magnitude. Increasing symptom severity corresponded with greater impairment across residual symptom-related nonwork activities, home management, ability to work, and social relationships. These relationships held when relevant covariates were included as well.

In support of prior research, insomnia patients experiencing residual symptoms comprise a group who are under particular strain, even relative to other already-burdened insomnia patients. This study uniquely describes the depth of this strain, which appears to occur across a wide range of domains and is likely affecting patients' global functioning and quality of life. Increasing residual symptom severity appears to affect level of impairment. Regarding financial burden, the strain could be indirectly affecting the work force and healthcare system. As hypothesized, older patients experiencing residual sleep medication effects showed the additional burden of more medical comorbidities. The comorbid conditions could potentially be aggravated or exacerbated by sleep medication side effects.

There are a number of limitations of the current study that should be considered alongside the results. Most importantly, this was an observational study, and the correlational nature of the data collection precludes any causal attribution. Likewise, the cross-sectional design does not allow us to ascertain whether the residual symptoms precede difficulties in home management, ability to work, and so forth, or whether residual symptoms occur in response to a worsening of such problems. Indeed, some residual symptoms, such as grogginess and difficulty concentrating, are also symptoms of insomnia, so some of the residual symptoms reported here may instead be symptoms of inadequately treated insomnia rather than next-day effects of sleep medication or a combination of both inadequate efficacy and medication side effects. Residual effects were self-reported rather than using objective measures of attention, memory, or reaction time. Another study limitation includes the margin of error inherent in any study using self-report measures, though insomnia itself can only be diagnosed via self-report, making self-report vital to this study [11]. Finally, the residual sleep medication effects we reported likely relate to other medical, psychosocial, quality of life, and economic outcomes that we did not measure. We may thus be underestimating the true extent of humanistic and economic burden.

## 5. Conclusions

Ultimately, patients who experience residual sleep medication effects represent a group with significant impairment of workplace, home, and social life activities; as the perceived severity of the residual symptoms increases, so does the burden. Thus, thorough medical and psychosocial/behavioral assessment of individuals experiencing residual effects is recommended (especially for the elderly). Also, improved management of insomnia would be beneficial. Behavioral and cognitive interventions have essentially no side effects and have been shown to lead to long-lasting, sustained improvements in sleep symptoms and parameters over 6 months to 24 months [28]. However, the degree of sleep medication use in this sample demonstrates that many may prefer, or need, pharmacotherapy for insomnia, highlighting a need for medications with fewer residual symptoms. The development of sleep medications with reduced residual effect profiles will be important for treatment of this patient population.

## Figures and Tables

**Table 1:** Respondent characteristics.

	Current user	
	*n*	%
Age (Mean, SD)	52.06	12.7
Age (10-year brackets)		
Under 25	27	1.7
25–34	148	9.2
35–44	256	16.0
45–54	429	26.7
55–64	472	29.4
65–74	238	14.8
75 and older	35	2.2
Female	1260	78.5%
Non-white	203	12.6%
Completed college	791	49.3%
Annual household income		
Below $25k	356	22.2%
$25–<50k	397	24.7%
$50–<75k	281	17.5%
$75k and above	481	30.0%
Decline to answer	90	5.6%
Employed	778	48.5%
BMI (Mean, SD)	26.6	6.6
BMI (categories)		
Underweight	39	2.4%
Normal	544	33.9%
Overweight	472	29.4%
Obese (up to 35)	248	15.5%
Obese (over 35)	200	12.5%
Decline to answer	102	6.4%
Alcohol use	1050	65.4%
Current smoker	387	24.1%
Exercise in previous month	1018	63.4%
Psychiatric comorbidities		
Alcoholism	60	3.7%
GAD or SAD	510	31.8%
Depression	802	50.0%
Schizophrenia	169	10.5%
Bipolar disorder	170	10.6%
Fibromyalgia	221	13.8%
Diagnosing doctor		
General Practitioner/Family Practitioner/Internist	1075	67.0
Psychiatrist	358	22.3
Sleep Specialist	103	6.4
Other	69	4.3
Prescribing doctor		
General Practitioner/Family Practitioner/Internist	1132	70.5
Psychiatrist	363	22.6
Sleep Specialist	33	2.1
Other	77	4.8
Current sleep medication		
Benzodiazepine	331	20.6%
Z-drug	809	50.4%
Antidepressant	335	20.9%
Other	130	8.1%
Still using first sleep medication	341	21.2%

**Table 2:** Respondent characteristics by presence of residual symptoms.

	Residual symptoms				*p* value
	None (*N* = 331)		One or more (*N* = 1,274)		
	*n*	%	*n*	%	
Age (Mean, SD)	54.1	12.7	51.5	12.6	0.001
Female	259	78.2%	1001	78.6%	0.898
Non-white	40	12.1%	163	12.8%	0.729
College degree	227	68.6%	862	67.7%	0.750
Annual household income					0.032
Below $25k	73	22.1%	283	22.2%	
$25–<50k	71	21.5%	326	25.6%	
$50–<75k	52	15.7%	229	18.0%	
$75 k and above	106	32.0%	375	29.4%	
Decline to answer	29	8.8%	61	4.8%	
Employed	153	46.2%	625	49.1%	0.358
BMI (Mean, SD)	27.1	6.8	28.0	6.6	0.048
CCI (Mean, SD)	0.60	1.11	0.77	1.36	0.032^*∗*^
Alcohol use	224	67.7%	826	64.8%	0.333
Current smoker	75	22.7%	312	24.5%	0.488
Exercise in previous month	207	62.5%	811	63.7%	0.706
Self-report psychiatric diagnoses					
Alcoholism	7	2.1%	53	4.2%	0.081
GAD or SAD	72	21.8%	438	34.4%	<0.001
Depression	128	38.7%	674	52.9%	<0.001
Schizophrenia	23	6.9%	146	11.5%	0.017
Bipolar disorder	26	7.9%	144	11.3%	0.069
Fibromyalgia	31	9.4%	190	14.9%	0.009
Diagnosing doctor for insomnia					0.002
General Practitioner/Family Practitioner/Internist	240	72.5%	835	65.5%	
Psychiatrist	50	15.1%	308	24.2%	
Sleep Specialist	21	6.3%	82	6.4%	
Other	20	6.0%	49	3.8%	
Prescribing doctor					0.005
General Practitioner/Family Practitioner/Internist	246	74.3%	886	69.5%	
Psychiatrist	54	16.3%	309	24.3%	
Sleep Specialist	11	3.3%	22	1.7%	
Other	20	6.0%	57	4.5%	

Note: *∗* indicates Welch's test was used in lieu of parametric *t*-test due to nonhomogeneity of variance.

**Table 3:** Sleep-related trouble according to the presence of residual symptoms.

	Residual symptoms				*p* value
	None (*N* = 331)		One or more (*N* = 1,274)		
	Mean	SD	Mean	SD	
Nights out of 7 with trouble falling asleep	5.1	2.3	5.3	2.0	0.297^*∗*^
Nights out of 7 with trouble staying asleep	5.4	2.2	5.4	2.2	0.856
Number of days out of 7 waking before alarm	4.6	2.6	4.5	2.5	0.253
Number of days out of 7 waking tired/unrested	4.6	2.4	5.6	1.9	<0.001^*∗*^
Nights out of 7 with any problem above	6.0	1.5	6.1	1.5	0.891
Sleep problems interfere with home management	3.9	3.0	5.6	2.8	<0.0001
Sleep problems interfere with ability to work	3.1	3.1	4.6	3.2	<0.0001
Sleep problems interfere with social relationships	3.6	3.2	5.3	3.0	<0.0001
Sleep problems interfere with close relationships	3.3	3.2	5.2	3.1	<0.0001

Note: *∗* indicates Welch's test was used in lieu of parametric *t*-test due to nonhomogeneity of variance.

**Table 4:** Respondent characteristics by experience of residual symptoms in respondents aged 65 and older.

	Residual symptoms				*p* value
	None (*N* = 78)		One or more (*N* = 195)		
	*n*	%	*n*	%	
Age (Mean, SD)	69.37	4.07	69.72	4.53	0.558
Female	63	80.8%	143	73.3%	0.197
Non-white	4	5.1%	8	4.1%	0.709
Completed college	59	75.6%	145	71.4%	0.466
Annual household income					0.076
Below $25k	13	16.7%	32	16.4%	
$25–<50k	13	16.7%	61	31.3%	
$50–<75k	14	17.9%	34	17.4%	
$75k and above	26	33.3%	53	27.2%	
Decline to answer	12	15.4%	15	7.7%	
Employed	15	19.2%	33	16.9%	0.651
BMI (categories)					0.968
Underweight	3	3.8%	6	3.1%	
Normal	26	33.3%	66	33.8%	
Overweight	28	35.9%	65	33.3%	
Obese (up to 35)	11	14.1%	34	17.4%	
Obese (over 35)	7	9.0%	19	9.7%	
Refused	3	3.8%	5	2.6%	
Alcohol use	51	65.4%	133	68.2%	0.653
Smokes	13	16.7%	23	11.8%	0.282
Exercise in previous month	43	55.1%	113	57.9%	0.671
Psychiatric comorbidities					
Alcoholic	1	1.3%	6	3.1%	0.397
Anxiety	8	10.3%	45	23.1%	0.016
Depression	18	23.1%	76	39.0%	0.013
Schizophrenia	1	1.3%	33	16.9%	0.000
Bipolar disorder	3	3.8%	5	2.6%	0.570
Fibromyalgia	4	5.1%	31	15.9%	0.016
Diagnosing doctor					0.074
General Practitioner/Family Practitioner/Internist	66	84.6%	142	72.8%	
Psychiatrist	5	6.4%	29	14.9%	
Sleep Specialist	2	2.6%	15	7.7%	
Other	5	6.4%	9	4.6%	
Prescribing doctor					0.264
General Practitioner/Family Practitioner/Internist	82.1%	162	83.1%	82.1%	
Psychiatrist	7.7%	24	12.3%	7.7%	
Sleep Specialist	3.8%	3	1.5%	3.8%	
Other	6.4%	6	3.1%	6.4%	

Note: *∗* indicates Welch's test was used in lieu of parametric *t*-test due to nonhomogeneity of variance.

**Table 5:** Sleep-related trouble according to the presence of residual symptoms in respondents 65 years and older.

	Residual symptoms				*p* value
	None (*N* = 78)		One or more (*N* = 195)		
	Mean	SD	Mean	SD	
Nights out of 7 with trouble falling asleep	4.6	2.5	5.2	2.1	0.059^*∗*^
Nights out of 7 with trouble staying asleep	5.4	2.2	5.4	2.2	0.822
Number of days out of 7 wake up before alarm	4.6	2.7	4.3	2.7	0.461
Number of days out of 7 wake up tired/unrested	3.7	2.7	4.9	2.4	0.001^*∗*^
Nights out of 7 with problem	5.9	1.6	5.8	1.8	0.651
Sleep problems interfere with home management	2.7	2.8	4.6	2.8	<0.001
Sleep problems interfere with ability to work	1.7	2.5	3.0	3.0	0.001^*∗*^
Sleep problems interfere with social relationships	2.6	3.0	3.9	2.8	0.001
Sleep problems interfere with close relationships	2.5	3.1	3.5	2.9	0.010

Note: *∗* indicates Welch's test was used in lieu of parametric *t*-test due to nonhomogeneity of variance.
